# A comprehensive analysis of biosorption of metal ions by macroalgae using ICP-OES, SEM-EDX and FTIR techniques

**DOI:** 10.1371/journal.pone.0205590

**Published:** 2018-10-15

**Authors:** Izabela Michalak, Małgorzata Mironiuk, Krzysztof Marycz

**Affiliations:** 1 Department of Advanced Material Technologies, Faculty of Chemistry, Wrocław University of Science and Technology, Wrocław, Poland; 2 Department of Experimental Biology, The Faculty of Biology and Animal Science, Wrocław University of Environmental and Life Sciences, Wrocław, Poland; 3 Faculty of Veterinary Medicine, Equine Clinic—Equine Surgery, Justus-Liebig-University, Gießen, Germany; University of Eastern Finland, FINLAND

## Abstract

In the present study, a comprehensive approach to the biosorption process was proposed. Biosorption of Cr(III), Mn(II) and Mg(II) ions by a freshwater macroalga *Cladophora glomerata* was examined using several advanced techniques including FTIR (Fourier Transform Infrared Spectroscopy), ICP-OES (Inductively Coupled Plasma–Optical Emission Spectrometry) and SEM-EDX (Scanning Electron Microscopy with Energy Dispersive X-Ray Spectroscopy). The enriched biomass can become a valuable, bioactive feed additive for different breeds of animals. Additionally, the collected algal biomass was soaked in water in order to reduce the content of carbohydrate, what is especially important for animals with metabolic disorders. The content of starch was reduced by 22% but additionally some elements–mainly Si, K and P were removed from the biomass. It was shown that the natural macroalga had better biosorption properties than soaked. Cr(III) ions were sorbed by the biomass in the highest extent, then Mn(II) and finally Mg(II) ions. The content of chromium in the enriched algal biomass increased almost ~200 000 times, manganese ~75 times and magnesium ~4.5 times (both for Mg(II) ions used from magnesium sulphate, as well as from magnesium chloride) when compared to the natural *Cladophora glomerata*. In the case of the soaked biomass the increase of the content of elements in the enriched biomass was as follows ~17 165 times for Cr, ~25 times for Mn and for Mg ~3.5 times for chloride and 3.8 times for sulphate. The type of magnesium salt (chloride or sulphate) had no significant effect on the algal sorption capacity. The proposed mechanism of the biosorption is ion exchange in which mainly potassium participated. The applied FTIR analysis enabled the identification of the functional groups that participated in the biosorption process–mainly carboxyl and hydroxyl. The main changes in the appearance of the spectra were observed for the following wavenumbers– 3300–3400; 2900; 1700; 1400–1500 and 1200–1300 cm^-1^. The application of SEM-EDX proved that the metal ions were sorbed on the surface of both tested algae.

## Introduction

Nowadays, the novel feeding strategies that will improve animals production, as well as their health are developed and implemented. A series of promising innovations and practices in feed production and feeding includes a balanced and phased feeding, the increase of the quality and level of use of forages in diets, reduction of grains use, a targeted mineral feeding etc. [1, 2]. These proposals are especially important in terms of horse feeding, where a creation of a well-balanced diet is of particular importance. A forage diet, low in non-structural carbohydrates, with a proper content of minerals and vitamins is highly recommended [3]. The second issue is to search for alternative/additional feed ingredients, mainly due to the global demand for grains and the competition between man and the livestock industry with the existing food and feeds [2]. There are several promising new feed resources, rich in biologically active compounds, that can be used as feed additives for animals. One of them is the biomass of algae, both micro- and macroalgae (also called seaweeds). In the literature, most of the attention is paid to marine macroalgae, however freshwater algae, being the result of eutrophication of water reservoirs, are also a rich source of biologically active compounds, such as unsaturated and saturated fatty acids, carbohydrates, amino acids and proteins, phenolic compounds, minerals, carotenoids, vitamins etc., and can constitute a very useful raw material for nutritional applications [3, 4]. The proposed approach is consistent with the concept of the Food and Agriculture Organization of the United Nations (FAO) of the sustainable animal diet, “which integrates the importance of efficient use of natural resources, protection of the environment, socio-cultural benefits and ethical integrity” [2].

In order to obtain a valuable feed additive with micro- and macroalements based on algal biomass, biosorption process can be applied. This is a surface phenomenon in which metal ions from aqueous solutions are passively bound by a non-living biomass [5–11]. Several operating conditions, such as solution pH, metal ions concentration, biomass size and its content in the solution, contact time, etc., influence the amount of metal ions bound with the biomass [8, 9, 11, 12, 13]. The choice of metal ion that will be sorbed by the biomass depends on the animal species and animal's requirement for a given nutrient.

The ability of the biomass to bind metal ions can be examined with the use of several advanced techniques such as FTIR, SEM-EDX and ICP-OES. In the available literature, such a comprehensive approach to the biosorption process is randomly presented, e.g., see in [12, 14, 15]). Among tested techniques, FTIR is the most often used in biosorption studies. According to the Web of Science database (accessed on 15^th^ May 2018), “FTIR” and “biosorption” appeared in the topic of the publications 1885 times, whereas in their title only 15 times. For “SEM” and “biosorption” it was 1634 and 7 times, respectively, for “SEM-EDX” and “biosorption”– 189 and 4 times, respectively and finally for “ICP” and “biosorption” 170 and 4 times, respectively. These techniques, used together, provide a full insight into biosorption process. FTIR is used to determine changes in the vibration frequency of functional groups of biosorbents before and after biosorption process, thus indicating which groups participated in biosorption [8, 9, 12, 13, 15, 16, 17]. ICP-OES is a precise and sensitive technique which allows to determine mineral composition of the whole natural and enriched biomass, after its digestion in mineral acids [6, 12, 14], whereas SEM-EDX enables determination of the elemental composition of the biomass surface and the distribution of metal ions [8, 9, 16, 12, 15, 17].

It is important to emphasize that the enriched with microelement ions algal biomass was tested both *in vitro* and *in vivo*. The enriched with Cr(III) ions freshwater macroalga *Cladophora glomerata* (*C*. *glomerata*) reduced apoptosis and inflammation in adipose derived mesenchymal stromal stem cells (ASCs) isolated from EMS horses (with Equine Metabolic Syndrome) through the improvement of mitochondrial dynamics, decreasing of PDK4 (Pyruvate Dehydrogenase Kinase 4) expression and reduction of the endoplastic reticulum stress. It was showed, that *C*. *glomerata* enriched with Cr(III) ions served antioxidative protection coming from enhanced SOD (Superoxide Dismutase) activity [18]. In *in vivo* tests which were conducted on pigs and laying hens it was shown that the algal biomass rich in microelements positively influenced the health of animals, their productivity, the quality of products (the content of micro- and macroelements), as well as the decrease of the excretion of elements what can lead to the decrease of the environmental burden [19–23].

The aim of the present paper was to obtain and characterise a feed additive for animals, especially for equine nutrition based on the biomass of a freshwater macroalga–*Cladophora glomerata*. The biomass was enriched separately with Cr(III), Mn(II) and Mg(II) ions, because they seem to be crucial in an equine clinical nutrition [3, 12, 18, 24]. In a dietary management of horses, especially with a diagnosed metabolic syndrome, soaking of hay in water is also recommended. It results in a significant reduction of the content of non-structural carbohydrates, e.g., simple sugars, starch and fructan [25, 26, 27]. Therefore, the algal biomass was also subjected to the soaking before biosorption process. The characteristics of the obtained feed additives was performed using ICP-OES, FTIR and SEM-EDX techniques.

## Materials and methods

### Biomass of freshwater macroalgae

*Cladophora glomerata* (*C*)–a freshwater macroalga was collected by hand from the surface of pond in a village Tomaszówek, Poland (51°27′21″N 20°07′43″E) twice–in August (*C*_A_) and October (*C*_O_) 2016. No specific permissions were required for this location/activity. The study was carried out on private land and the owner of the land gave permission to conduct the study on this site. These studies did not involve endangered or protected species. Then, the air-dried biomass was milled using a grinding mill (Retsch GM300, Haan, Germany). For the biosorption experiments, the biomass with the particle size lower than 500 μm was chosen.

### Soaking of *Cladophora glomerata*

For the soaking, 800 g of a dried *Cladophora glomerata* (*C*_A_; August 2016) was suspended in 40 L of tap water for 72 h. After this period, it was filtered and the air-dried soaked *Cladophora* biomass (S*C*_A_) was used in biosorption.

### Biosorption process

**Fig 1:**
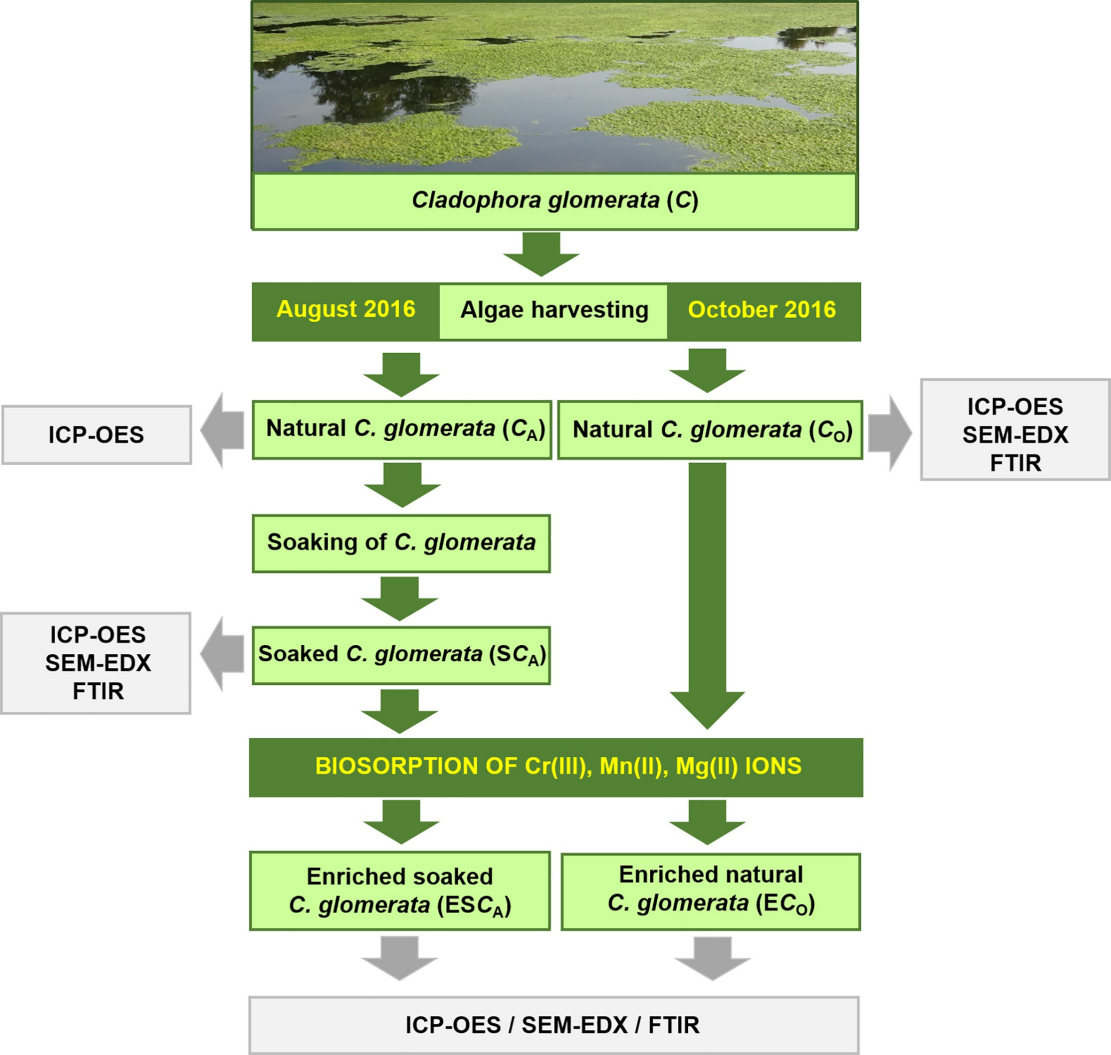
A general scheme of performed experiments.

The biosorption process of Mn(II), Cr(III) and Mg(II) ions was performed in Erlenmeyer flasks containing 1 g of the biomass (the natural collected in October 2016 –*C*_O_ and soaked–S*C*_A_) and 500 mL of the metal ions solution in a shaker (IKA KS 260 basic) at 150 rpm in room temperature. The concentration of each metal ion was 200 mg/L. The solutions were prepared in deionized water by dissolving separately, appropriate amounts of inorganic salts–MnSO_4_·H_2_O, Cr(NO_3_)_3_·9H_2_O, MgCl_2_·6H_2_O; MgSO_4_·7H_2_O (POCh S.A. Gliwice, Poland). pH 5 of solutions was adjusted with 0.1 mol/L solutions of NaOH/HCl (POCh S.A. Gliwice, Poland) using a pH meter Mettler-Toledo (Seven Multi; Greifensee, Switzerland) equipped with an electrode InLab413 with the compensation of temperature. The contact time was 3 hours determined in our previous experiments [12]. A general scheme of preformed experiments is presented in Fig 1.

### Analytical techniques

#### Chemical composition of macroalgae used in experiments

The natural *Cladophora glomerata* collected in August 2016 (*C*_A_), as well as the soaked biomass (S*C*_A_) were examined in terms of their nutritional value. Protein was determined according to PB-142 I edition (14.05.2012), fibre–PN-EN ISO 6865:2002, ash–PN-R-64795:1976, fat–GAFTA 3:0 edition 2014, starch–HEC81-3.

#### ICP-OES technique

The natural (*C*_O_ and *C*_A_), soaked (S*C*_A_), as well as enriched with metal ions biomass (E*C*_O_ and ES*C*_A_) was mineralized with nitric acid– 0.5 g of biomass and 5.0 mL of 69% HNO_3_ (Suprapur, Merck KGaA, Darmstadt, Germany) in a microwave oven StartD (Milestone MLS-1200 MEGA, Bergamo, Italy) and analyzed using ICP-OES spectrometer (Varian VISTA-MPX ICP-OES, Victoria, Australia). Analyses were performed in the Chemical Laboratory of Multielemental Analysis at Wrocław University of Science and Technology, which is accredited by International Laboratory Accreditation Cooperation Mutual Recognition Arrangement and Polish Centre for Accreditation (No AB 696).

#### SEM-EDX technique

The natural (*C*_O_), soaked (S*C*_A_), as well as enriched with metal ions biomass (E*C*_O_ and ES*C*_A_) was analyzed using SEM-EDX technique. For a morphological observation, SEM was applied according to the previously described method [6]. The samples of the biomass were fixed in 2.5% of glutaraldehyde (Sigma) and were dehydrated by ethanol (from 30% till 100% concentration). Next, samples were covered with gold using a sputter coater (Scancoat six, Oxford) and observed in a scanning electron microscope (EVO LS15, ZEISS, Oberkochen, Germany) operating at 15 kV. The elemental analysis, as well as ions distribution (mapping) on investigated samples were performed using BRUCKER energy dispersive X-ray system according to the previously published methodology [28, 29]. The X-ray spectrum of each *Cladophora glomerata* loaded with a given microelement was obtained.

#### FTIR analysis

Before analysis, samples of the natural (*C*_O_, S*C*_A_) and enriched biomass of algae (E*C*_O_ and ES*C*_A_) were dried for 24h at 80°C. For FTIR analysis, KBr discs were prepared containing 1.5 mg of the algal sample and 200 mg of KBr. The spectra were recorded on a Bruker spectrophotometer (Bruker FT-IR IFS 66/s; Billerica, Massachusetts, USA) in the mid IR range (4000–400 cm^-1^).

## Results

This manuscript provides a comprehensive insight into biosorption properties of a freshwater macroalga–*Cladophora glomerata*, natural and additionally soaked in tap water before this process. The biomass was enriched separately with Cr(III), Mn(II) and Mg(II) ions in a batch system. The initial concentration of metal ions in the aqueous solution (*C*_0_) was 200 mg/L, pH 5, the biomass content (*C*_S_) was 2 g/L and contact time 3h. The experimental conditions were established in our previous study [12]. After biosorption, the enriched biomass was examined using several techniques such as ICP-OES, FTIR and SEM-EDX. As a control group, the natural and soaked biomass was used. A nutritional value of the natural and soaked *Cladophora glomerata* was also determined.

### The nutritional value of the natural and soaked *Cladophora glomerata*

The treatment of metabolic disorders in animals requires a proper diet, which should be low in non-structural carbohydrates. Soaking in water components of forage can partially remove them [26, 27]. In the present study we wanted to check how soaking of macroalgae in water affects their nutritional value. The obtained results are presented in Table 1. It is important to note that the soaking of freshwater macroalgae in tap water resulted in the reduction of starch by 22% in the soaked macroalga when compared with the natural one.

**Table 1:** A nutritional value of the natural and soaked *Cladophora glomerata*.

Parameter	Natural *C*. *glomerata* (*C*_A_) *	Soaked *C*. *glomerata* (S*C*_A_) *
**Protein (%)**	22.5	21.7
**Fiber (%)**	19.6	21.6
**Ash (%)**	22.6	21.8
**Fat (%)**	0.4	0.2
**Starch (%)**	4.1	3.2

* for a particle size lower than 500 **μ**m

In our previous study we showed that the freshwater *Cladophora glomerata* collected in August 2016 was a rich source of biologically active compounds and contained vitamin C (21.8±0.5 mg/100 g of algal dry mass (d.m.)), α-tocopherol (2.4±0.3 mg/100 g of d.m.), polyphenols (196±8 mg/100 g of d.m.), free and protein-bound amino acids (e.g., Ala, Glc, Val, Leu, Ile, Asn, Asp, Gln, Glu, Lys, Arg, His, Phe, Tyr, Trp, Ser, Thr, Met, Cys, Pro) and fatty acids [18]. Fatty acids present in the biomass of *C*. *glomerata* are listed in Table 2. In the work of Messyasz et al. (2015) it was shown that the extracts obtained with ethanol and acetone from a freshwater *Cladophora* also contained some of the mentioned fatty acids, for example: C12:0, C14:0, C16:0, C18:0, C16:1 (*n*-7), C18:2 (*n*-6), C18:3 (*n*-3), C18:4 (*n*-3) [4].

**Table 2:** Content of fatty acids in a natural *Cladophora glomerata* [17].

Fatty acid	Name	Content (mg/100 g d.m.)
C8:0	caprylic acid	0.50±0.0
C10:0	capric acid	0.30±0.10
C12:0	lauric acid	0.23±0.06
C14:0	myristic acid	83.8±0.5
C14:1	*n*-5; myristoleic acid	0.50±0.05
C15:0	pentadecylic acid	1.0±0.2
C16:0	palmitic acid	160±1.0
C16:1	*n*-7; palmitoleic acid	30.0±0.1
C18:0	stearic acid	4.8±0.6
C18:1	*n*-12; petroselinic acid	33.5±0.3
C18:2	*n*-6; linoleic acid	14.2±0.4
C18:3	*n*-3; alpha-linolenic acid; ALA	23.0±1.1
C18:3	*n*-6; gamma-linolenic acid; GLA	not detected
C18:4	*n*-3; stearidonic acid	29.7±0.6
C20:0	arachidic acid	0.80±0.10
C20:2	*n*-6; eicosadienoic acid	not detected
C22:0	behenic acid	4.0±0.7

The nutritional value of *Cladophora glomerata* can vary depending on the conditions in which it grows. Khuantrairong and Traichaiyaporn (2011) present a composition of a freshwater alga *Cladophora* sp. grown in the laboratory under different phosphorus concentrations. It was found that *Cladophora* sp. contained averagely protein: 10.7–17.7%; fibre: 20.7–26.1%; ash: 14.7–16.9%; lipids; 2.0–2.6% and carbohydrate: 52.5–61.0%. Authors indicated that protein content increased under high phosphorus concentration, while carbohydrate content decreased [30]. The tested in the present study *Cladophora glomerata*, grown in the natural freshwater environment, was a richer source of proteins and ash. Moreover, it was found that the soaking of the algal biomass in water reduced the content of starch by 22%. Longland et al. (2011) also showed that the soaking of hay reduced the content of carbohydrate, whereas crude protein was not leached from the hay during soaking [25]. Our research showed that the content of protein decreased by 4% in the soaked alga. This issue is very important taking into account a proper feeding of horses with metabolic disorders, which should not be fed with high-starch concentrates [3].

### ICP-OES technique in biosorption process

ICP-OES is a precise and sensitive analytical technique with low detection limits. It enables determination of elements in the whole biomass, not only on the surface as it is in the case of SEM-EDX. However, it must be proceeded by the biomass mineralization with concentrated inorganic acids. This technique is used for the analysis both solutions, as well as the biomass before and after biosorption [6].

In the biosorption process, examined metal ions from the aqueous solution are exchanged with metal ions which are naturally bound with functional groups on the surface of the biomass cell wall. Therefore, the dominating mechanism of biosorption is ion exchange [5, 6, 9, 10, 31]. The untreated biomass generally contains light metal ions such as K, Na, Ca and Mg which are acquired from the water [5, 9]. In this paper we wanted to check if the freshwater biomass of *Cladophora glomerata* is able to bind light metal ions, for example magnesium. This element has never been used in the biosorption studies as a sorbate. Moreover, magnesium was chosen since is thought to elicit its positive effect on insulin sensitivity what is very important in the case of laminitic obese horses [32]. Additionally we checked if there is an effect of the magnesium inorganic salt (sulphate and chloride) used for the preparation of a stock solution for the biosoption process on its sorption by algae. In the literature it is noted that metal anions (e.g., sulphate, nitrate, chloride) can influence the biosorption capacity of green algae [33].

In Table 3, the multielemental composition of the natural biomass of *Cladophora glomerata* (*C*_O_) before and after biosorption (E*C*_O_-Cr (as nitrate), E*C*_O_-Mg (as sulphate), E*C*_O_-Mg (as chloride), E*C*_O_-Mn (as sulphate)) determined by ICP-OES is presented.

**Table 3:** A multielemental composition of the natural biomass of *Cladophora glomerata* (*C*_O_)* before and after biosorption determined by ICP-OES (mg/kg d.m.).

Element and wavelength	Natural *C*. *glomerata* (*C*_O_)	Enriched *C*. *glomerata*			
		E*C*_O_-Cr (as nitrate)	E*C*_O_-Mg (as sulphate)	E*C*_O_-Mg (as chloride)	E*C*_O_-Mn (as sulphate)
Al 308.215	263.5±39.5	229.2±34.4	249.2±37.4	301.2±45.2	327.2±49.1
B 249.772	45.52±6.83	24.16±3.63	57.94±8.69	58.40±8.76	51.03±7.65
Ba 455.403	87.72±13.16	60.05±9.01	86.32±12.9	89.38±13.41	88.18±13.23
Ca 315.887	148 927±29 784	49 509±9 902	110 490±22 098	113 373±22 675	109 708±21 942
Cd 228.802	0.06264±0.01253	0.03931±0.00786	0.03626±0.00725	0.03440±0.00688	0.01567±0.003134
Cr 267.716	0.4346±0.0652	**86 614**±17 323	6.285±0.943	5.238±0.786	6.937±1.040
Cu 324.754	3.652±0.548	2.475±0.371	4.250±0.637	4.340±0.651	3.786±0.568
Fe 259.940	541.3±81.2	619.1±92.9	664.0±99.6	649.5±97.4	667.0±100.0
K 766.491	20 643±4 129	838.8±125.8	1 383± 277	1 202±240	1 347±269
Mg 285.213	1 748±350	431.4±65.7	**7 981**±1 596	**7 679**±1 536	836.0±125.4
Mn 257.610	123.0±18.4	80.86±12.13	92.21±13.83	99.35±14.90	**9 227**±1 845
Na 588.995	647.1±97.1	288.1±43.2	287.4±43.1	241.1±36.2	161.2±24.2
Ni 231.604	2.920±0.438	9.599±1.440	7.230±1.084	6.437±0.966	2.359±0.354
P 213.618	947.2±142.1	1 356±271	727.3±109.1	657.6±98.6	900.6±135.1
Pb 220.353	1.774±0.231	<LOD	2.644±0.344	2.783±0.362	1.297±0.169
S 181.972	13 415±2 683	13 660±2 732	17 639±3 528	14 718±2 944	15 979±3 196
Si 251.611	635.9±95.4	26.41±3.96	24.31±3.65	38.26±5.74	31.12±4.67
Zn 213.857	19.21±2.88	52.59±7.89	52.64±7.90	54.49±8.17	46.94±7.04

< LOD–below a limit of detection; bold–the biomass enriched with a given element

* for a particle size lower than 500 **μ**m

Comparing the content of Cr, Mg and Mn in the natural and in the enriched biomass it can be noticed that the content of chromium increased in the enriched biomass by about 200 000 times when compared with the natural, Mn increased by about 75 times and Mg about 4.4 times when it was used as a chloride and about 4.6 times when Mg was used as a sulphate. In the case of chromium biosorption, main metal ions which participated in the ion exchange and were removed from the biomass to the aqueous solution were: K, Si, Ca, Mg and Na. After biosorption, the content of K decreased ~25 times in the enriched biomass, when compared to the natural, Si ~24 times, Mg ~4 times, Ca ~3 times and Na ~2.2 times.

Magnesium which was sorbed by macroalga from solutions prepared from magnesium sulphate and chloride showed similar properties. The decrease of the content of elements in the enriched biomass when compared to the natural, concerned the following micro- and macroelements: Si decreased by ~26 times, K ~15 times, Na ~2.2 times, Ca ~1.4 times–for Mg used as a sulphate salt and Si decreased by ~17 times, K ~17 times, Na ~2.7 times, Ca ~1.3 times–for Mg used as a chloride salt.

The biosorption of manganese resulted in the release mainly of Si, which was ~20 times lower in the enriched when compared to the natural biomass, K was ~15 times lower, Na was ~4 times lower, Mg was ~2.1 times lower and Ca was ~1.4 times lower. From the results it can be noted that beside light metal ions–alkali metal cations (Na, K) and alkaline earth metals (Mg, Ca), also silicon participated in this process. This element is known to be a nutrient required for the growth of *Cladophora glomerata*, but also an essential component of the algal cell wall [34].

Table 4 presents a multielemental composition of a natural *Cladophora glomerata* (collected in August 2016 –*C*_A_) which was later soaked for 48 hours (S*C*_A_) and finally enriched with Cr(III)*–*ES*C*_A_-Cr, Mg(II)–ES*C*_A_-Mg (separately as magnesium sulphate and magnesium chloride) and Mn(II) ions–ES*C*_A_-Mn.

**Table 4:** A multielemental composition of the natural and soaked biomass of *Cladophora glomerata* (S*C*_A_)* and the enriched soaked alga determined by ICP-OES (mg/kg d.m.).

Element and wavelength	Natural *C*. *glomerata* (*C*_A_)	Soaked *C*. *glomerata* (S*C*_A_)	Enriched soaked *C*. *glomerata*			
			ES*C*_A_-Cr (as nitrate)	ES*C*_A_-Mg (as sulphate)	ES*C*_A_-Mg (as chloride)	ES*C*_A_-Mn (as sulphate)
Al 308.215	561.8±84.3	672.6±100.9	720.7±108.1	847.2±127.1	784.1±117.6	747.7±112.2
B 249.772	126.5±19.0	146.3±21.9	112.4±16.9	162.3±24.3	158.8±23.8	158.5±23.8
Ba 455.403	54.93±8.24	72.87±10.93	42.11±6.32	67.52±10.13	64.22±9.63	70.31±10.55
Ca 315.887	56 786±11 357	50 748±10 150	18 918±3 784	48 691±9 738	45 019±9 004	49 723±9 945
Cd 228.802	0.1030±0.0134	< LOD	0.4521±0.0588	0.2366±0.0308	0.2249±0.0292	0.1396±0.0181
Cr 267.716	6.157±0.924	3.718±0.558	**63 819**±12 764	4.523±0.678	4.781±0.717	14.35±2.15
Cu 324.754	4.898±0.735	7.703±1.155	5.255±0.788	5.783±0.867	2.914±0.437	5.670±0.850
Fe 259.940	1 650±330	1 645±329	2 024±405	1 899±380	1 904±381	2 031±406
K 766.491	25 122±5 024	8 551±1 710	286.8±43.0	516.5±77.5	453.8±68.1	496.8±74.5
Mg 285.213	2 951±590	2 339±468	414.8±62.2	**8 869**±1 774	**8 275**±1 655	717.2±107.6
Mn 257.610	588.6±88.3	476.8±71.5	224.8±33.7	338.6±50.8	329.3±49.4	**11 954**±2 391
Na 588.995	723.4±108.5	817.1±122.6	727.8±109.2	212.5±31.9	108.3±16.2	90.66±13.60
Ni 231.604	2.143±0.321	6.925±1.039	4.985±0.748	8.694±1.304	10.12±1.52	15.77±2.37
P 213.618	2 269±454	1 174±235	1 332±266	978.4±146.8	1 005±201	1 125±225
Pb 220.353	2.760±0.359	2.816±0.366	2.302±0.299	4.763±0.619	7.497±0.975	7.187±0.934
S 181.972	20 912±4 182	19 308±3 862	17 236±3 447	19 305±3 861	17 155±3 431	18 877±3 775
Si 251.611	821.3±123.2	15.78±2.37	9.592±1.439	20.30±3.04	19.77±2.97	20.03±3.00
Zn 213.857	15.96±2.39	54.19±8.13	49.20±7.38	55.31±8.30	50.40±7.56	55.21±8.28

< LOD–below a limit of detection; bold–the biomass enriched with a given element

* for a particle size lower than 500 **μ**m

The results showed that the biomass soaking in tap water led to the removal of some elements. The main differences were obtained mainly for Si, where its content in the soaked biomass was ~52 times lower than in the natural biomass, for K the decrease was ~2.9 times, P ~1.9 times, for Mg decrease was by 21% and for Mn by 19%.

Comparing the content of Cr, Mg and Mn in the soaked and in the enriched biomass it can be noticed that the content of chromium increased by about 17 165 times in the enriched biomass when compared to the soaked, Mn increased ~25 times and Mg increased ~3.5 times when it was sorbed as a magnesium chloride and increased ~3.8 times when it was sorbed as a magnesium sulphate. During the biosorption of Cr(III) ions, Ca, K and Mg were the main elements which were released from the soaked biomass to the aqueous solution (due to cation exchange). The content of K decreased ~30 times in the enriched biomass, when compared to the soaked biomass before biosorption, Mg decreased ~5.6 times, Ca ~3 times, Mn ~2.1 times and Si ~1.6 times. Biosorption of Mg(II) ions in the form of sulphate and chloride salts by the soaked *C*. *glomerata* showed similarities. The main metal ions which were released from the biomass during biosorption of Mg(II) ions were K, which content in the enriched biomass decreased by ~16.6 times (for sulphate) and ~18.8 times (for chloride) when compared to the soaked biomass before biosorption, Na decreased by ~3.8 times (for sulphate) and ~7.5 times (for chloride), Mn by ~29% (for sulphate) and ~31% (for chloride) and P by ~17% (for sulphate) and ~14% (for chloride). During biosorption of Mn(II) ions, in the cation exchange participated mainly K–a decrease of the content in the enriched soaked biomass by ~17.2 times when compared to the soaked biomass before biosorption), Na–a decrease by ~9.0 times and Mg–a decrease by ~3.3 times.

In this study it was also found that the best biosorption properties showed the biomass of the natural, as well as the soaked *Cladophora glomerata* towards Cr(III), then Mn(II) and finally Mg(II) ions. The extent to which a metal ion will be bound to functional groups on the surface of biomass cell wall largely depends on the chemistry of the sorbed metal ion (oxidation state, electrochemical potential, ionization potential, electronegativity, atomic radius, ionic radius, atomic weight, atomic number, covalent index etc., [9, 35, 36]) and its preference to form bonds [36]. The most important parameters of metal ion are a covalent index (the greater the covalent index value of a metal ion, the greater its potential to form covalent bonds with biological ligands) and ionization potential [35, 36]. In the case of *Cladophora glomerata*, the amount of metal ions bound with the biomass increased (Cr > Mn > Mg) with the increase of a covalent index (X_m_^2^*r*, where X_m_ is electronegativity and *r*–a ionic radius): Cr– 1.71, Mn– 1.61, Mg– 1.24 and the ionization potential: Cr (30.96) > Mn (15.64) > Mg (15.03).

The binding of Cr(III), Mn(II) and Mg(II) ions by the algal biomass was associated with the ion exchange with mainly K(I), Na(I), Ca(II) for all tested ions and additionally with Mg(II) for Cr(III) and Mn(II) ions biosorption. For both macroalgae–natural (*C*_O_) and soaked (S*C*_A_), potassium was the main light metal ion that participated in the ion exchange in biosorption process (Tables 3 and 4). The content of K decreased in the highest extent among other light metal ions in the enriched biomass, when compared to the raw material used in the biosorption process. This is in accordance with the literature data. Pouya and Behnam (2017) based on ICP analysis suggested that the ion exchange can be considered as a main mechanism for copper removal by a red alga–*Jania adhaerens*, because ions of Na (considerably higher than other ions), K, B, Al and Fe were released from the biomass into the solution upon biosorption process [15]. Also Dmytryk et al. (2014) showed the replacement of naturally bound by *Spirulina* sp. light metal ions: Na(I), K(I), or Ca(II) with biosorbed microelements such as Co(II), Cu(II), Mn(II) and Zn(II) [14].

Additionally it was presented that for both tested algae–natural (*C*_O_) and soaked (S*C*_A_), the type of magnesium salt (sulphate/chloride) did not influence significantly the sorption of Mg(II) by the biomass. Slightly higher content of magnesium in the enriched biomass was noted for the sulphate. Similar results were obtained for a green marine macroalga–*Enteromorpha prolifera*, where biosorption capacity of Cu(II) ions used as chloride was 46.0 mg/g, whereas for copper sulphate it was 42.8 mg/g [33].

In the present research it was also shown that soaking of algae in water decreased the content of mainly K, P, Mg, Mn in the biomass. The ash content in a soaked *C*. *glomerata* was by 4% lower than in the raw material (Table 1). This is in agreement with the literature data that indicates that the soaking of hay significantly reduced the level of sodium, potassium, magnesium, phosphorus and copper (but presumably not nitrogen, calcium, zinc, manganese and iron which were also tested) [37].

### The effect of the season on the multielemental composition of freshwater macroalgae

**Fig 2:**
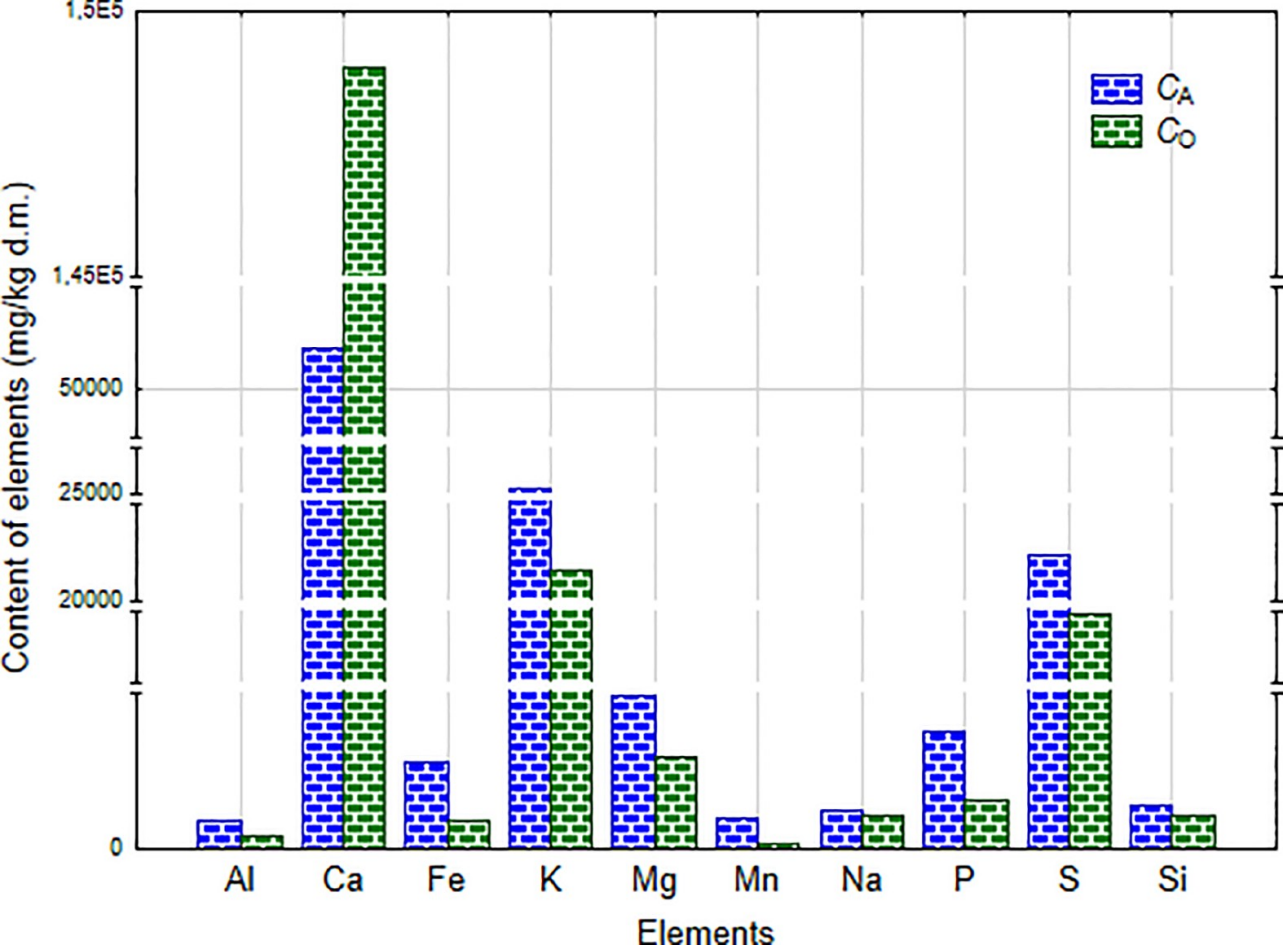
A comparison of a multielemental composition of a natural *Cladophora glomerata* collected in August (*C*_A_) and in October 2016 (*C*_O_).

In this paper we also examined the effect of the season on the multielemental composition of freshwater macroalgae. The results from ICP-OES analysis showed that the content of elements in freshwater macroalgae depended on the season–it was higher in summer (August) than in autumn (October)–Fig 2.

*Cladophora glomerata* collected in August was a richer source of most of the elements than *C*. *glomerata* collected in October, for example contained ~4.8 times more of Mn, ~3 times more of Fe, ~2.4 times more of P, by ~69% more of Mg, by ~56% more of S, by ~23% more of Si, by ~22% more of K and by ~12% more of Na. Only the content of Ca in algal biomass collected in October was much more higher (~3 times) than in alga from August. The mineral composition of algae depends on many factors, for example the type of algae species, the place of their harvest (ocean, sea, pond, river etc.,) and its geography, residence time in a water reservoir and other seasonal, annual, environmental and physiological factors [4, 38]. In the work of Michalak et al. (2009) it was shown that a green macroalga *Enteromorpha* sp. collected from the Gulf of Gdańsk (Poland) in June (summer) contained higher amounts of Mn, Mg, Ca, K and Fe than the alga collected in April (spring–lower temperatures) [38]. For the future research it will be beneficial to collect algae mainly in the summer season because they are a richer source of micro-and macroelements than algae harvested in the autumn, especially taking into account their potential application as feed additves with elements for animals. Additionally, after biosorption of metal ions, the enriched biomass can constitute a concentrated form of micro- and macronutrients. Moreover, the summer is the most favourable season for algae growth what ensures their availability.

### Scanning electron microscopy (SEM) combined with energy dispersive X-ray (EDX) in biosorption process

The SEM-EDX investigation allows the examination in details the surface concentration and distribution of Cr(III), Mn(II) and Mg(II) ions in the natural and enriched *Cladophora glomerata*. In our and other previous research it was shown, that SEM-EDX becomes a valuable tool for a precise investigation of the elemental composition, as well as distribution of elements in different biomasses [6, 18, 39]. In the present study, the SEM-EDX elemental analysis revealed the increased quantity of Mg, Cr, as well as Mn in the course of biosorption process in *Cladophora glomerata* biomass (Table 5).

**Table 5:** The weight percentage (*wt* %, mean±SD) of elements in the natural and soaked biomass of Cladophora glomerata* before and after biosorption determined by SEM-EDX.

Element	*Natural C*. *glomerata* (*C*_O_)		Soaked *C*. *glomerata* (S*C*_A_)	
	before biosorption	after biosorption	before biosorption	after biosorption
**Mg (as sulphate for biosorption)**	0.46±0.13	1.66±0.17 **	0.31±0.01	0.81±0.18 **
**Mg (as chloride for biosorption)**		0.46±0.09 ***		0.46±0.15 ***
**Cr(III) (as nitrate for biosorption)**	0.07±0.01^a^	4.78±1.17^a^	0.68±0.01	2.07±1.14
**Mn(II) (as sulphate for biosorption)**	0.18±0.05	1.56±0.70	0.42±0.13	3.20±1.84

* for a particle size lower than 500 **μ**m

**Mg as sulphate for biosorption

***Mg as chloride for biosorption

^a^ [12]

**Fig 3:**
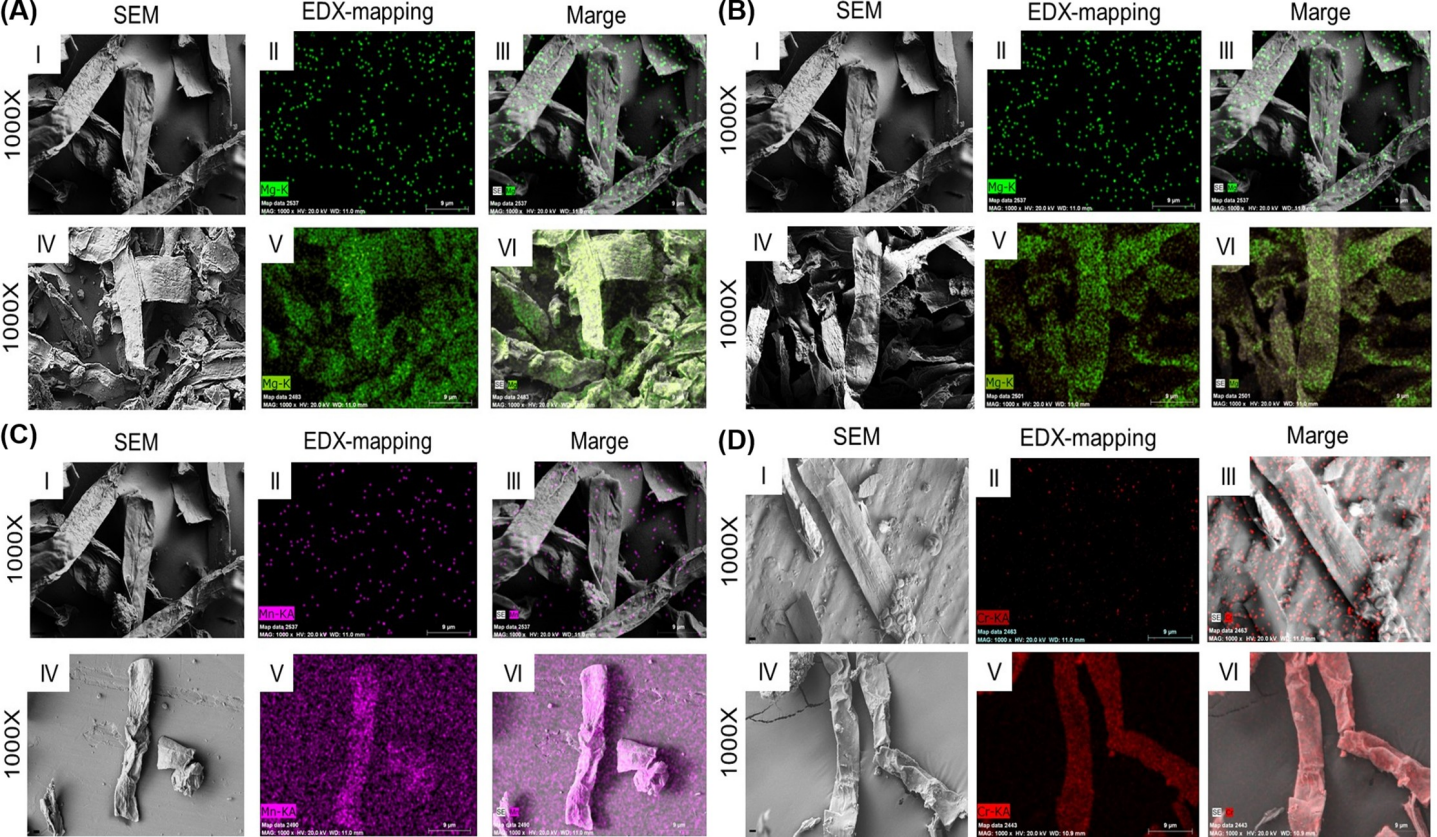
SEM-EDX images of the natural and enriched natural macroalga. (A) natural *Cladophora glomerata* (*C*_O_) before biosorption process (I, II, III) and enriched with Mg(II) ions as sulphate (IV, V, VI), (B) natural *C*. *glomerata* (*C*_O_) before biosorption process (I, II, III) and enriched with Mg(II) ions as chloride (IV, V, VI), (C) natural *C*. *glomerata* (*C*_O_) before biosorption process (I, II, III) and enriched with Mn(II) ions as sulphate (IV, V, VI) and (D) natural *C*. *glomerata* (*C*_O_) before biosorption process (I, II, III) and enriched with Cr(III) ions as nitrate [12] (IV, V, VI).

**Fig 4:**
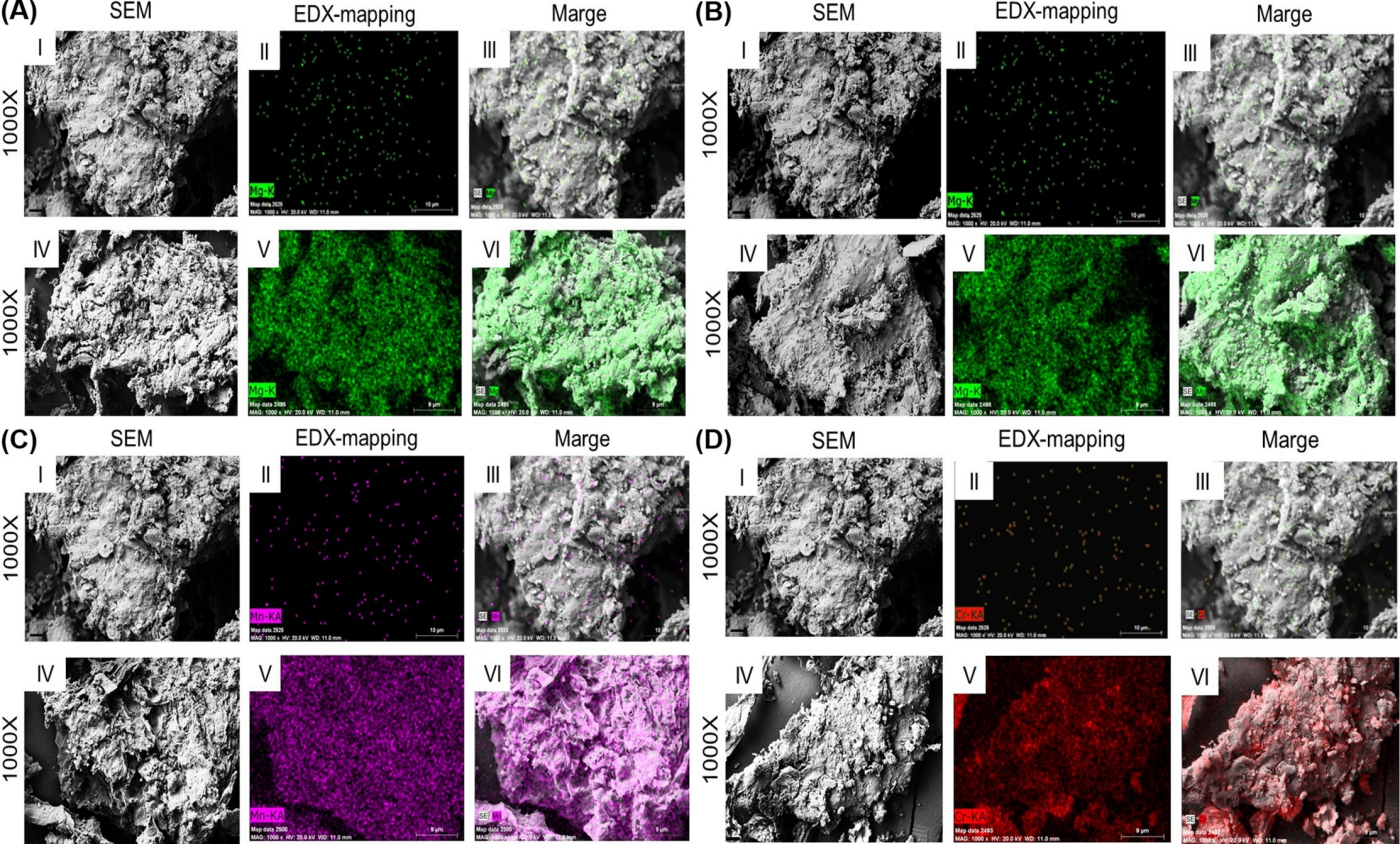
SEM-EDX images of the soaked and enriched soaked macroalga. (A) soaked *Cladophora glomerata* (S*C*_A_) before biosorption process (I, II, III) and enriched with Mg(II) ions as sulphate (IV, V, VI), (B) soaked *C*. *glomerata* (S*C*_A_) before biosorption process (I, II, III) and enriched with Mg(II) ions as chloride (IV, V, VI), (C) soaked *C*. *glomerata* (S*C*_A_) before biosorption process (I, II, III) and enriched with Mn(II) ions as sulphate (IV, V, VI) and (D) soaked *C*. *glomerata* (S*C*_A_) before biosorption process (I, II, III) and enriched with Cr(III) ions as nitrate (IV, V, VI).

The biosorption process resulted in the increased amount of Mg, Cr, as well as Mn in both soaking and non-soaking biomass what is shown in Figs 3 and 4, when compared to the native form of *Cladophora glomerata*. However, the non-soaking form of the investigated biomass was characterized by higher biosorption properties in the case of Cr(III) and Mg(II) ions. This can be probably due to the removal of native elements from algal biomass during soaking process, what finally could have reduced the biosorption properties of the soaked algal biomass.

The EDX mapping clearly showed, that Cr was equally distributed on the surface of the investigated biomass in the both soaking and non-soaking form (Figs 3 and 4D). It was found, that the soaking process negatively affected biosorption properties of *Cladophora glomerata* in the case of Cr(III) and Mg(II) ions, but surprisingly enhanced absorption of Mn(II) ions. The decreased quantity of Mg was observed in the biomass, that was soaked before biosorption process. The EDX mapping confirmed the equal surface distribution of Mg and Mn on the investigated *Cladophora glomerata*, regardless it was soaked or not (Figs 3 and 4).

In this study it was shown, that the algal biomass is characterized by good biosorption properties and can actively bind to its surface particular elements, including Cr(III), Mn(II) and Mg(II). In our research, it was demonstrated that among all the tested elements, Cr(III) ions were sorbed in the highest extent and chromium content in a non-soaking form was equal to 4.78±1.17%, when compared to the soaking form (2.07±1.14%). Using EDX method it was also demonstrated that all tested ions were successfully bound to the surface of *Cladophora glomerata*, what was shown using a mapping technique.

### FTIR analysis of the natural and enriched biomass of algae

**Fig 5:**
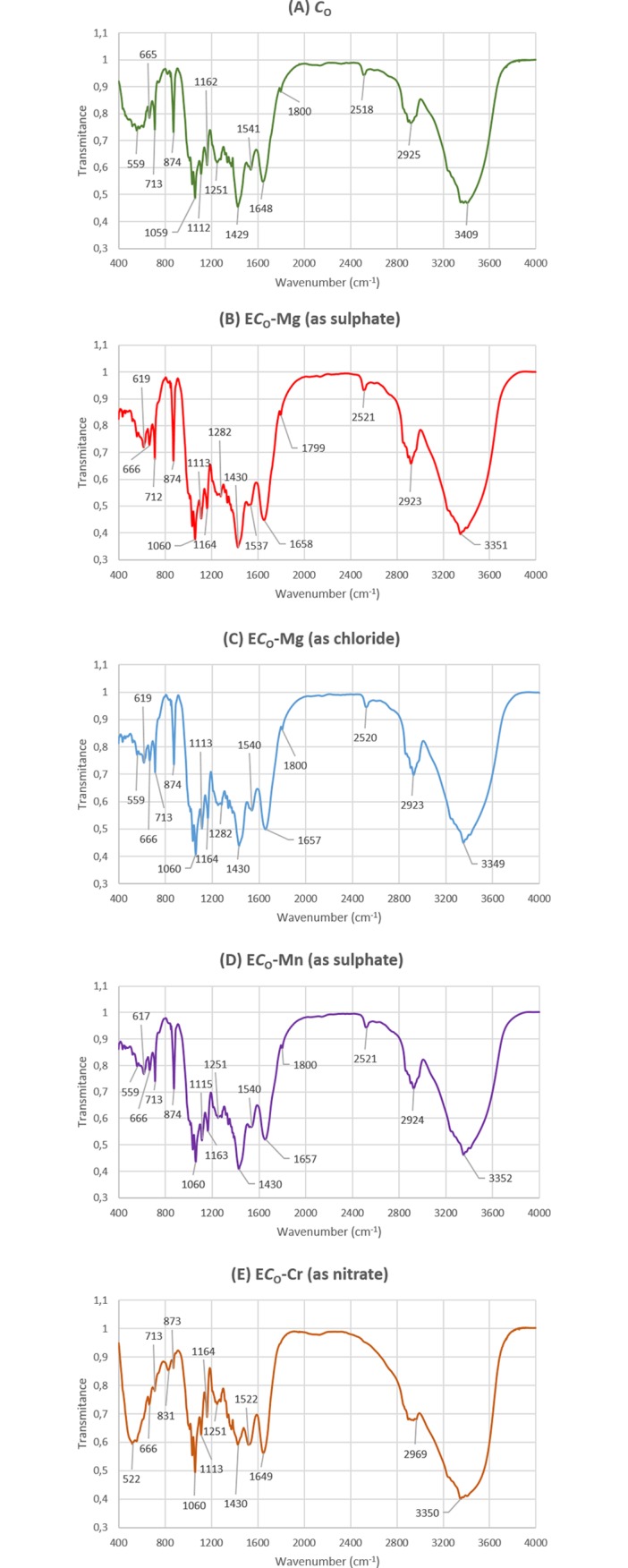
FTIR spectra of the natural and enriched natural macroalga. (A) natural *Cladophora glomerata* (*C*_O_) before biosorption process, (B) natural *C*. *glomerata* (*C*_O_) enriched with Mg(II) ions (E*C*_O_-Mg as sulphate), (C) natural *C*. *glomerata* (*C*_O_) enriched with Mg(II) ions (E*C*_O_-Mg as chloride), (D) natural *C*. *glomerata* (*C*_O_) enriched with Mn(II) ions (E*C*_O_-Mn as sulphate) and (E) natural *C*. *glomerata* (*C*_O_) enriched with Cr(III) ions (E*C*_O_-Cr as nitrate).

**Fig 6:**
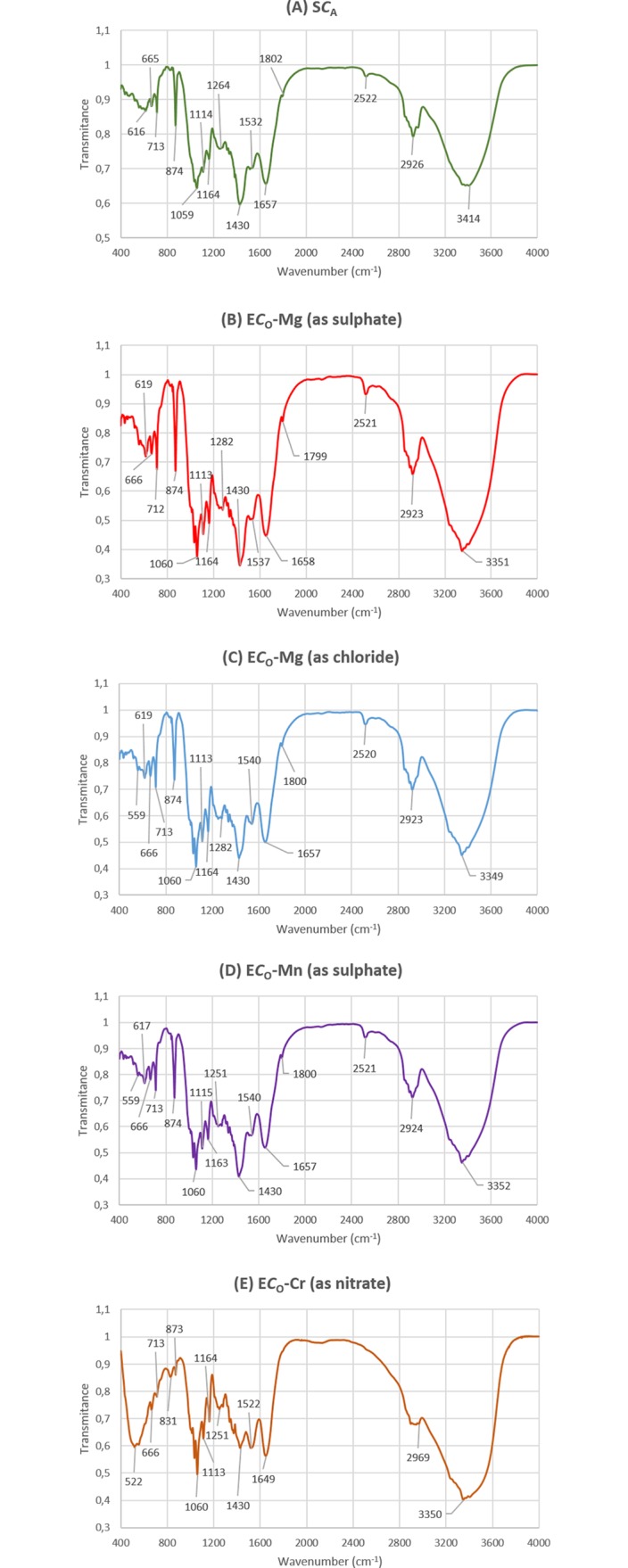
FTIR spectra of the soaked and enriched soaked macroalga. (A) soaked *Cladophora glomerata* (S*C*_A_) before biosorption process, (B) soaked *C*. *glomerata* (S*C*_A_) enriched with Mg(II) ions (ES*C*_A_-Mg as sulphate), (C) soaked *C*. *glomerata* (S*C*_A_) enriched with Mg(II) ions (ES*C*_A_-Mg as chloride), (D) soaked *C*. *glomerata* (S*C*_A_) enriched with Mn(II) ions (ES*C*_A_-Mn as sulphate) and (E) soaked *C*. *glomerata* (S*C*_A_) enriched with Cr(III) ions (ES*C*_A_-Cr as nitrate).

FTIR technique was used to examine a surface characterization of the biosorbent and to observe the chemical modification after biosorption of metal ions by *Cladophora glomerata*. Analysis of the natural and enriched biomass enables identification of the functional groups that participate in the biosorption process. These groups originate from macromolecules (polysaccharides, proteins and lipids) which are a part of the algal cell wall. The cell wall offers several functional groups such as carboxyl (e.g., fatty acid and amino acids), hydroxyl (e.g., polysaccharides), amine, phosphate or sulfonate etc., [14, 31]. FTIR spectra of the natural (*C*_O_) and soaked (S*C*_A_) *Cladophora glomerata*, as well as enriched with Mn(II), Mg(II) and Cr(III) ions were obtained in the region of 4000–400 cm^-1^ and are presented in Figs 5 and 6.

Table 6 presents a FTIR description of groups according to their wavelengths for the natural and soaked *Cladophora glomerata* before and after biosorption process.

**Table 6:** FTIR description for the natural and enriched *Cladophora glomerata*.

Wavenumber (cm^-1^) for the natural *C*. *glomerata* (*C*_O_)	The origin of the band	Wavenumber (cm^-1^) for the enriched *C*. *glomerata*			
		*C*_O_–Cr (as nitrate)	*C*_O_–Mg (as sulphate)	*C*_O_–Mg (as chloride)	*C*_O_–Mn (as sulphate)
3100–3700	stretching vibrations of O-H (polysaccharides) and stretching of N-H (proteins)	3100–3700	3100–3700	3100–3700	3100–3700
2850–2970	stretching vibrations of C-H of aliphatic groups CH_2_ and CH	2850–2970	2850–2970	2850–2970	2850–2970
2511	overtone band CO_3_^2-^/HCO_3_^-^ (2ν_2_ *+* ν_4_)	2511	2511	2511	2511
1799	overtone band CO_3_^2-^/HCO_3_^-^ (ν_1_ *+* ν_4_)	1799	1799	1799	1799
1652*	*bending vibrations of O-H of adsorbed water molecules*, *I amide band (proteins)*	*1652*	*1652*	*1652*	*1652*
1643**	bending vibrations of O-H of adsorbed water molecules, I amide band (proteins)	1647***	**1654**	**1654**	**1660**
1520	II amide band (proteins)	1520	1520	1520	1520
1450	in-plane bending vibrations of O-H	1450	1450	1450	1450
1428	symmetric bending vibrations of CH_2_, symmetric stretching vibrations of CO_3_ ions	1428	1428	1428	1428
1385*	*stretching asymmetric vibrations of the ion NO*_*3*_^*-*^ *(ν*_*2*_*)*	*1385*	*1385*	*1385*	*1385*
-	stretching asymmetric vibrations of the ion NO_3_^-^ (ν_2_)	-	**1385**	**1385**	**1385**
1370	in-plane bending vibrations of C-H	1370	1370	1370	1370
1362	in-plane bending vibrations of C-H	1362	1362	1362	1362
1338	in-plane bending vibrations of O-H	1338	1338	1338	1338
1320	bending vibrations of pyranose ring and wagging of CH_2_	1320	1320	1320	1320
1200–1280	bending vibrations of C-H and O-H, rocking vibrations of CH_2_ (polysaccharides), III amide band (proteins)	1200–1280	1200–1280	1200–1280	1200–1280
1161, 1115, 1160, 1130, 1012, 1005, 990	stretching vibrations of C-O and C-C, rocking vibrations of CH_2_, bending vibrations of a ring, stretching symmetric vibrations of the ions CO_3_^2-/^HCO_3_^-^ (ν_1_)	1161, 1115, 1160, 1130, 1012, 1005, 990	1161, 1115, 1160, 1130, 1012, 1005, 990	1161, 1115, 1160, 1130, 1012, 1005, 990	1161, 1115, 1160, 1130, 1012, 1005, 990
895	bending vibrations of C-O-C, C-OH, C-H of ring	895	895	895	895
872	out-of-plane bending vibrations of ions CO_3_^2-^/HCO_3_^-^ (ν_2_)	872	872	872	872
847	out-of-plane bending vibrations of ionsNO_3_^-^ (ν_2_)	-	847	847	847
711	in-plane bending vibrations of ions CO_3_^2-^/HCO_3_^-^ (ν_4_)	711	711	711	711
667	out-of-plane bending vibrations of C-OH	667	667	667	667
831, 745, 616, 559, 520, 430, 438	a series of non-specific bands associated with bending vibrations	831, 745, 616, 559, 520, 430, 438	831, 745, 616, 559, 520, 430, 438	831, 745, 616, 559, 520, 430, 438	831, 745, 616, 559, 520, 430, 438
~ 550	a broad band coming from the water's librational (swaying) band of water molecules	~ 550	~ 550	~ 550	~ 550

For the soaked *C*. *glomerata* (S*C*_A_), wavenumbers and the origin of the band were the same as for the natural *C*. *glomerata* (*C*_O_)–for a particle size lower than 500 **μ**m

* new wavenumbers and descriptions for the soaked *C*. *glomerata* (S*C*_A_) were marked in *Italics* (not present in the natural *C*. *glomerata–C*_O_)

** lack of this wavenumber in the soaked *C*. *glomerata* (S*C*_A_)

*** main differences in the wavenumbers between the natural/soaked *C*. *glomerata* and enriched

The FTIR spectra of natural and soaked algae before and after biosorption show a complex nature of the biomass due to the presence a large number of peaks. The broadening and shifting of peaks was observed after interaction of a raw biomass with the examined metal ions. The largest differences in the appearance of the spectrum were observed between the natural/soaked *Cladophora glomerata* and enriched with chromium ions. In Fig 5 it can be seen, that the spectra for the natural and enriched algae generally have similar shape, however especially in the case of E*C*_O_-Cr the increase in the transmittance of peaks in a chromium loaded biomass when compared to the metal free biomass is observed in the region ~1000–2000 cm^-1^ and decrease in the region ~2400–3600 cm^-1^. For biomasses: E*C*_O_-Mg as sulphate and chloride and E*C*_O_-Mn, transmittances of peaks in metal loaded biomasses are generally lower than in the unloaded biomass. In the case of the soaked *Cladophora glomerata* (S*C*_A_), the transmittance of peaks in the region ~1100–2000 cm^-1^ was smaller than for the loaded with Mn(II), Mg(II) (sulphate/chloride) and Cr(III) biomass and in the region of ~2400–3600 cm^-1^ with the exception of ES*C*_A_-Cr (Fig 6).

Main differences between the tested natural and enriched biomass were observed for the wavenumber range from approximately 3300–3400 cm^-1^ (overlapping of amine N–H and hydroxyl O–H stretching vibrations, especially in E*C*_O_-Cr and E*C*_O_-Mg (sulphate)), at ~2900 cm^-1^ for all enriched biomasses (–CH stretching vibration of C–CH_3_), at ~1700 cm^-1^ (C = O stretching mode of the amide I band), at ~1400–1500 cm^-1^ (stretching of C = O bond from carboxylic acids), in the range 1200–1300 cm^-1^ (bending vibrations of C-H and O-H, rocking vibrations of CH_2_ (polysaccharides), III amide band (proteins)) (Table 6). From Figs 5 and 6, it can be seen that transmittance values were shifted mainly for carboxyl and hydroxyl groups. It was also noted that differences in the transmittance of peaks of the unloaded and loaded biomass were higher for the natural *Cladophora glomerata*, (*C*_O_) rather than for soaked (S*C*_A_), what is in agreement with results obtained by ICP-OES.

In this study it was shown that hydroxyl and carboxyl groups (~ 1200–1280 cm^-1^; ~1400–1500 cm^-1^) were effective for capturing Cr(III), Mg(II) and Mn(II) ions (Figs 5 and 6). This is in line with results obtained in the previous work–biosorption of Cr(III) ions by *Vaucheria* sp. [31] and *Cladophora glomerata* [12]. Deng et al. (2007) also indicated from FTIR analysis that amino or hydroxy, C = O, and C–O groups of *Cladophora fascicularis* could combine strongly with Cu(II) ions [40].

## Conclusions

Summarizing, in the present research, we investigated biosorption properties of *Cladophora glomerata* in a native, as well as in a soaked form and then enriched with Cr(III), Mn(II) and Mg(II) ions. We chose these ions, because they are recognized as insulin sensitizers, which in turn in the form of the enriched biomass can serve as a potential clinical feed additive for horses that suffer from EMS. *Cladophora glomerata* showed the best biosorption properties towards Cr(III) ions, then Mn(II) and finally Mg(II) ions. In both macroalgae–natural and soaked, potassium was the main light metal ion that participated in the ion exchange in biosorption process. FTIR analysis showed that carboxyl and hydroxyl groups were effective for capturing Cr(III), Mg(II) and Mn(II) ions. SEM-EDX technique confirmed binding of metal ions on the surface of algal biomass. Our research clearly showed, that soaked *Cladophora glomerata* was characterized by the lower ability to bind Cr(III), Mn(II) and Mg(II) ions. Additionally it was found that soaking of algae in water decreased the content of mainly K, P, Mg, Mn in the biomass. Although, biosorption properties of a soaked *Cladophora glomerata* are reduced, when compared to the native form, it can serve as a low glycaemic component for the production of advanced feed supplements for horses (soaking of algae resulted in the reduction of starch by 22% when compared with natural macroalga). Moreover, the additional advantage of low glycaemic index components in particular produced from *Cladophora glomerata* might be the possibility to increase its quantity in a daily ration, which in turn will serve the higher amounts of bioactive factors. To summarize, it might be highlighted, that soaking of *Cladophora glomerata* negatively affected its biosorption properties, but reduced the glycemic index, which is crucial in the feeding of insulin resistance animals.
